# Longitudinal monitoring for the emergence of epidermal growth factor C797S resistance mutations in non-small cell lung cancer using blood-based droplet digital PCR

**DOI:** 10.20517/cdr.2019.53

**Published:** 2019-09-19

**Authors:** Mark Bowling, Hyder Arastu, Victoria Edwards, Leisa Jackson, Scott Thurston, Jordan Reese, Hestia Mellert, Gary Pestano, Paul Walker

**Affiliations:** ^1^East Carolina University, Mail Stop 628, Internal Medicine - Hematology/Oncology, Greenville, NC 27834, USA.; ^2^Biodesix, Inc., 2970 Wilderness Place, Boulder, CO 80301, USA.

**Keywords:** Epidermal growth factor, non-small cell lung cancer, C797S, drug resistance

## Abstract

Validation of assays for the C797S mutation as a biomarker for osimertinib resistance is promising in guiding treatment decision-making for multidrug resistant non-small cell lung cancer. A newly developed droplet digital PCR (ddPCR) assay was used to retrospectively evaluate the emergence of the C797S mutation in six remnant plasma samples in this case report. It was found that the detected emergence of C797S clearly correlated with clinical signs of treatment resistance. Had these data been available to aid treatment selection in real time, there would have been hope for recaptured disease response and control instead of treatment cessation. The results of this study show that highly sensitive ddPCR methods can be used for the monitoring of emergent epidermal growth factor somatic variant mutations in circulation.

## Introduction

The third-generation epidermal growth factor (EGFR) tyrosine kinase inhibitor (TKI) osimertinib is approved for front-line use as well as for use as a second-line treatment when EGFR-mutant, non-small cell lung cancer (NSCLC) becomes resistant to erlotinib, afatinib or gefitinib. This resistance is most frequently mediated by the T790M point mutation^[1]^. Unfortunately, acquired osimertinib resistance, considered to be associated with the C797S point mutation in many cases, can emerge within about 10 months in a high proportion of patients^[2]^. While no formal clinical study has yet validated the utility of the C797S mutation as a biomarker for osimertinib resistance, such information could guide treatment decision-making for multidrug resistant NSCLC.

### Analytic controls

#### Synthetic controls

Synthetic gene fragments were synthesized as gBlocks by Integrated DNA Technologies (Coralville, Iowa). The sequences for EGFR were designed so that each single nucleotide variant was represented by a unique gBlock. Lyophilized gBlocks were suspended in TE buffer to a stock concentration of 10 ng/µL and were further diluted to working concentrations as needed. Synthetic DNA oligos used to qualify the instrument and to assess initial analytic sensitivity and specificity were used at between 1 attogram and 1 femtogram concentrations per well as input into the respective droplet digital PCR (ddPCR) reactions.

### Recovery of cfDNA from plasma

Whole blood samples were prospectively collected into Cell-Free DNA (cfDNA) BCT® (Streck) tubes. The whole blood was processed to circulating cfDNA following standard operating procedures in the laboratory as previously published^[3]^. Briefly, whole blood samples were shipped overnight to the Biodesix CLIA Laboratory and processed to plasma first by centrifugation at low speed (1900 × *g* for 10 min at 4 ºC), followed by a high speed spin of the plasma fraction (16,000 × *g* for 10 min at 4 ºC). cfDNA was isolated using the QIAamp Circulating Nucleic Acid Kit (Qiagen) according to standard operating procedure. DNA was quantified by Qubit dsDNA HS Assay Kit (Life Technologies/Thermo Fisher). cfDNA was used at 2.5 ng per reaction in the ddPCR workflow.

### ddPCR

cfDNA specimens were retrospectively analyzed using ddPCR. ddPCR reactions (PrimePCR^TM^; BioRad) were prepared in duplicate. Each 20 µL reaction contained the following: 10 µL of 2× ddPCR Supermix for probes (no dUTP), 1 µL of EGFR C797S multiplex assay^[4]^, 7 µL of the test template DNA (cfDNA), and 2 µL nuclease-free water. No template control reactions were performed with water in place of the DNA template and were run with each assay within a plate.

Droplet generation was performed with a manual droplet generation QX200 system (BioRad) following standard operating procedures. Once emulsions were generated, plates were placed into a C1000 Touch thermal cycler (BioRad). The thermal cycling profile was performed as follows: 95 ºC, 10 min (enzyme activation, 1 cycle) followed by denaturation (94 ºC, 30 s) and annealing/extension (55 ºC, 1 min), ramp rate of ~2 ºC/s; 40 cycles. To conclude the procedure, enzyme deactivation was done at 98 ºC, 10 min followed by hold at 4 ºC (ramp rate of ~1 ºC/s). After amplification, the plate was transferred to the droplet reader (BioRad). Samples were read using the Rare Event Detection module on the reader (QuantaSoft version 1.7.4.0917).

### Data analysis, review and result generation

Data review and analysis were conducted to determine negative and positive droplet counts for each sample using the QuantaSoft analysis modules for calculating mutant and wild-type copy numbers. The wild-type DNA variants were used as assay quality controls to verify the presence of circulating nucleic acids of sufficient quality and quantity for testing. The ddPCR cut-off values were set based on visual review of the signal amplitudes for positive and negative (no template) control samples. The DNA-variant test (EGFR) results were either expressed as number of copies or by the percent minor variant frequency (%MVF) of the mutation in relation to wildtype. The clinical cut-off for calling a positive sample in the validation studies was defined at 0.02% MVF. Variant frequencies were calculated as follows:

%MVF = (Mutation Copy Number)/(Mutation + WT Copy Number)*100

## Case report

In October 2014, a never-smoker female presented with new-onset seizures and was diagnosed with an extensive brain and bony metastatic TTF-1 positive lung adenocarcinoma. Tissue EGFR exon 19 deletion (del19) was identified. After an excellent initial response to whole-brain radiation therapy and ongoing erlotinib, she was switched to osimertinib when T790M resistance emerged in July 2016. Molecular testing was conducted using the validated GeneStrat ddPCR platform (Biodesix, Inc., Boulder, Colorado USA) for EGFR L858R, del19 (E746-A750), and T790M^[3]^. By January 2018 (osimertinib day 540) the patient had discontinued therapy due to resistance and disease progression. She died in hospice care at age 46.

**Figure 1:**
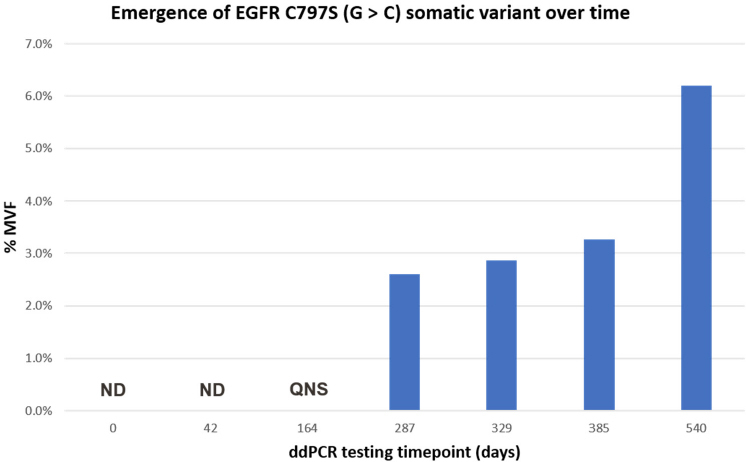
Kinetics of emergence of the C797S (G>C) somatic mutant variant during osimertinib treatment cycle. Results are shown as %MVF. For timepoints 0 and 42 C797S was ND. Although results were obtained for del19 and T790M for the day-164 specimen, the remaining specimen QNS for C797S testing at this time point. ND: not detected; QNS: quantity was not sufficient; %MVF: percent minor variant frequency

**Figure 2:**
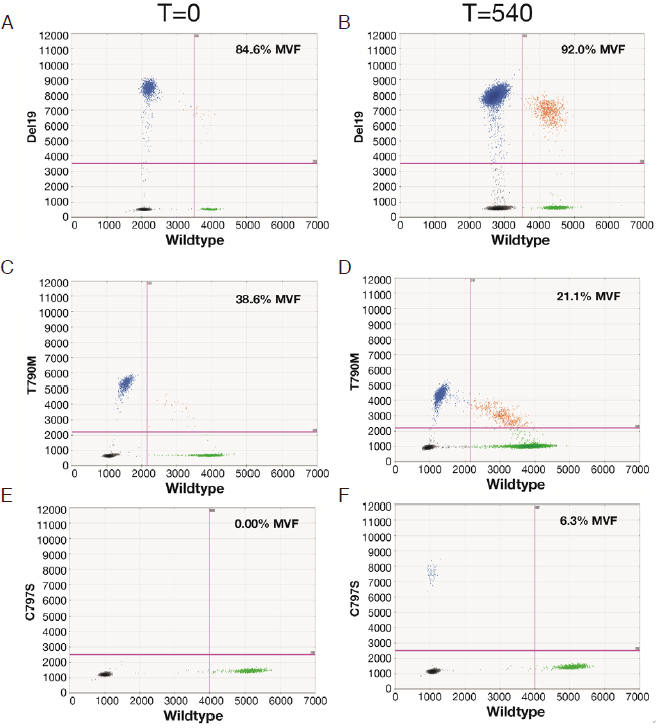
Evolution of EGFR del19, T790M, and C797S during the 540-day osimertinib treatment cycle. Data are displayed as relative fluorescence units, and the calculated %MVF values are shown for each test. A and B: Del19 on days 0 and 540, respectively; C and D: T790M on days 0 and 540, respectively; E and F: C797S on days 0 and 540, respectively. EGFR L858R was consistently found to be negative by both ddPCR and NGS tests (data not shown). EGFR: epidermal growth factor; %MVF: percent minor variant frequency; ddPCR: droplet digital PCR; NGS: next-generation sequencing

**Figure 3:**
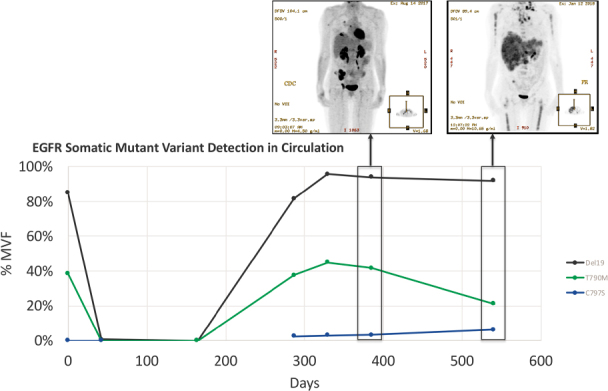
Detection of del19 driver and T790M TKI resistance mutations compared to C797S osimertinib resistance mutation (%MVF) over 540-day osimertinib treatment cycle. Note the day-164 specimen was unavailable for testing for C797S because the sample was exhausted. Insets: patient PET-CT scan on days 385 and 540 (showing disease progression). EGFR: epidermal growth factor; %MVF: percent minor variant frequency; TKI: tyrosine kinase inhibitor

We used a newly developed ddPCR assay to retrospectively evaluate the emergence of the C797S mutation in six remnant plasma samples from this patient. All Biodesix personnel were blinded to patient case information. The C797S mutation was undetectable on treatment days 0 and 42 but was detectable by day 287 [Figure 1]. Orthogonal testing with next-generation sequencing showed that only the C797S variant (G>C) was present in circulation. The EGFR del19 and T790M mutations both declined after initial osimertinib treatment but re-emerged by day 287 [Figures 2 and 3].

## Discussion

As hypothesized, the detected emergence of C797S mutations correlated with clinical signs of treatment resistance. Interestingly, while signals for the del19 driver mutation and T790M resistance mutations were detected on initiation of osimertinib therapy (July 2016), and subsequently declined (August through December 2016), both re-emerged and with greater detectable signal than C797S [Figure 3]. However, while the detection of the del19 variant reached a steady state and T790M decreased as disease progressed, the levels of the C797S variant in circulation continued to rise. We hypothesize that these results indicate the emergence, over time, of a clonal C797S variant sub-population. It is noteworthy that while the C797S mutation allele frequency increased at later timepoints, the T790M frequency declined across those same timepoints. There could be several reasons for this observation. One possibility is that osimertinib treatment results in specific killing of T790M only cells, which contributes to the decline in detectable T790M signal, but not for C797S. Additionally, the kinetics of death and active nucleic acid shedding by cells are complex and are mostly for monitoring purposes at this time. In any event, given this patient’s young age, had these data been available to aid treatment decision in real-time and based on the cis/trans nature of the T790M and C797S mutation, there might have been an opportunity to implement a first-generation TKI, with hope for recaptured disease response and control, instead of treatment cessation^[5]^.

The results of this study, using blinded case specimens, show that highly sensitive ddPCR methods can be used for the monitoring of emergent EGFR somatic variant mutations in circulation. In this case study, we have demonstrated that this method has potential clinical utility for detecting and monitoring osimertinib treatment resistance via the C797S escape mechanism.

## Declarations

### Copyright

© The Author(s) 2019.
